# Age-related twin-peak prevalence profiles of H. pylori infection, gastritis, GIN and gastric cancer: Analyses of 70,534 patients with gastroscopic biopsies

**DOI:** 10.1371/journal.pone.0265885

**Published:** 2022-07-21

**Authors:** Meng Qing Xu, Ke Sun, Chong Cao, Hui Hui Yin, Xiao Jun Wang, Qi Hang Yin, Li Jie Wang, Lin Tao, Kui Wang, Feng Li, Wen Jie Zhang

**Affiliations:** 1 Department of Pathology, The First Affiliated Hospital, Shihezi University School of Medicine, Shihezi, Xinjiang, China; 2 The Key Laboratory for Xinjiang Endemic and Ethnic Diseases, Shihezi University School of Medicine, Shihezi, Xinjiang, China; 3 Department of Gastroenterology, Jinling Hospital, Nanjing, Jiangsu, China; 4 Department of Preventive Medicine, Shihezi University School of Medicine, Shihezi, Xinjiang, China; 5 Department of Pathology, Beijing Chaoyang Hospital, Capital Medical University, Beijing, China; University of Malaya Faculty of Medicine, MALAYSIA

## Abstract

**Objectives:**

H. pylori (Hp) infection has been indicated in the pathogenesis of gastric diseases including gastric cancer (GC). This study aimed at exploring the relationships between Hp infection and gastric diseases including GC in a large dataset of routine patients undergoing gastroscopy.

**Methods:**

From November 2007 to December 2017, 70,534 first-time visiting patients aged 18–94 years with gastroscopic biopsies were histologically diagnosed and analyzed. Patients’ data were entered twice in an Excel spreadsheet database and analyzed using the SPSS (version 22.0) software package and statistical significance was defined as P<0.05 for all analyses.

**Results:**

The first interesting observation was age-related twin-peak prevalence profiles (TPPs) for Hp infection, gastritis, and advanced diseases with different time spans (TS) between the first and second occurring peaks. Hp infection and gastritis had TPPs occurring at earlier ages than TPPs of gastric introepithelial neoplasia (GIN) and GC. More patients were clustered at the second occurring TPPs. The time spans (TS) from the first occurring peak of Hp infection to the first occurring peaks of other gastric diseases varied dramatically with 0–5 years for gastritis; 5–15 years for GINs, and 5–20 years for GC, respectively. The number of males with Hp infection and gastric diseases, excluding non-atrophic gastritis (NAG), was more than that of females (P<0.001).

**Conclusions:**

We have first observed age-related twin-peak prevalence profiles for Hp infection, gastritis, GIN, and GC, respectively, among a large population of patients undergoing gastroscopy. The second prevalence peak of GC is at ages of 70–74 years indicating that many GC patients would be missed during screening because the cut-off age for screening is 69 years old in China.

## Introduction

Gastric cancer (GC) is very common in China which poses a great threat to the public health [1, 2]. In recent years, the morbidity and mortality of GC have been stabilized, however, the situation at large remains severe. Esophagogastroduodendoscopy (EGD) is the paramount method for diagnosis of GC [3] with a high sensitivity of 60% - 80% [4]. It is of great significance for the early diagnosis and early treatment of GC.

Correa cascade [5–7] refers to the development from non-atrophic gastritis to atrophic gastritis, then to intestinal metaplasia, and finally to atypical hyperplasia, which is usually considered to be a common developing order for intestinal non-cardiac gastric cancer [8]. Moreover, H. pylori (Hp) infection has been generally accepted as the initiator of this cascade [9] and the main pathogenic factor of chronic gastritis, peptic ulcer, gastric mucosa-associated lymphoid tissue, and gastric cancer [5, 10]. In 1994, the International Agency for Research on Cancer (IARC) classified Hp as a class I carcinogen [11]. For the past few years, numerous studies have found a clear correlation between Hp infection and GC [12, 13], which is still a hot topic in research.

Therefore, the exploration of the changing trend of Hp infection and gastric diseases, as well as their relationships with age is of great significance for the prevention and control of GC. Due to the lack of big data analysis in this field, it is still unclear about the pathogenesis of GC and the related Hp infection, Gastritis and GIN, bringing great challenges to early diagnosis and early treatment. Based on histologically diagnosed gastroscopic biopsies derived from 70,534 cases, we have analyzed: (1) the relationships between Hp infection and gastric diseases, including GC, in a large dataset of gastroscopic patients; (2) the dynamic trends of Hp infection, gastric diseases, and their relationships with age and the age-related prevalence profiles for gastric diseases; (3) optimal ages for screening of gastric diseases.

## Methods

### Patients

**Fig 1:**
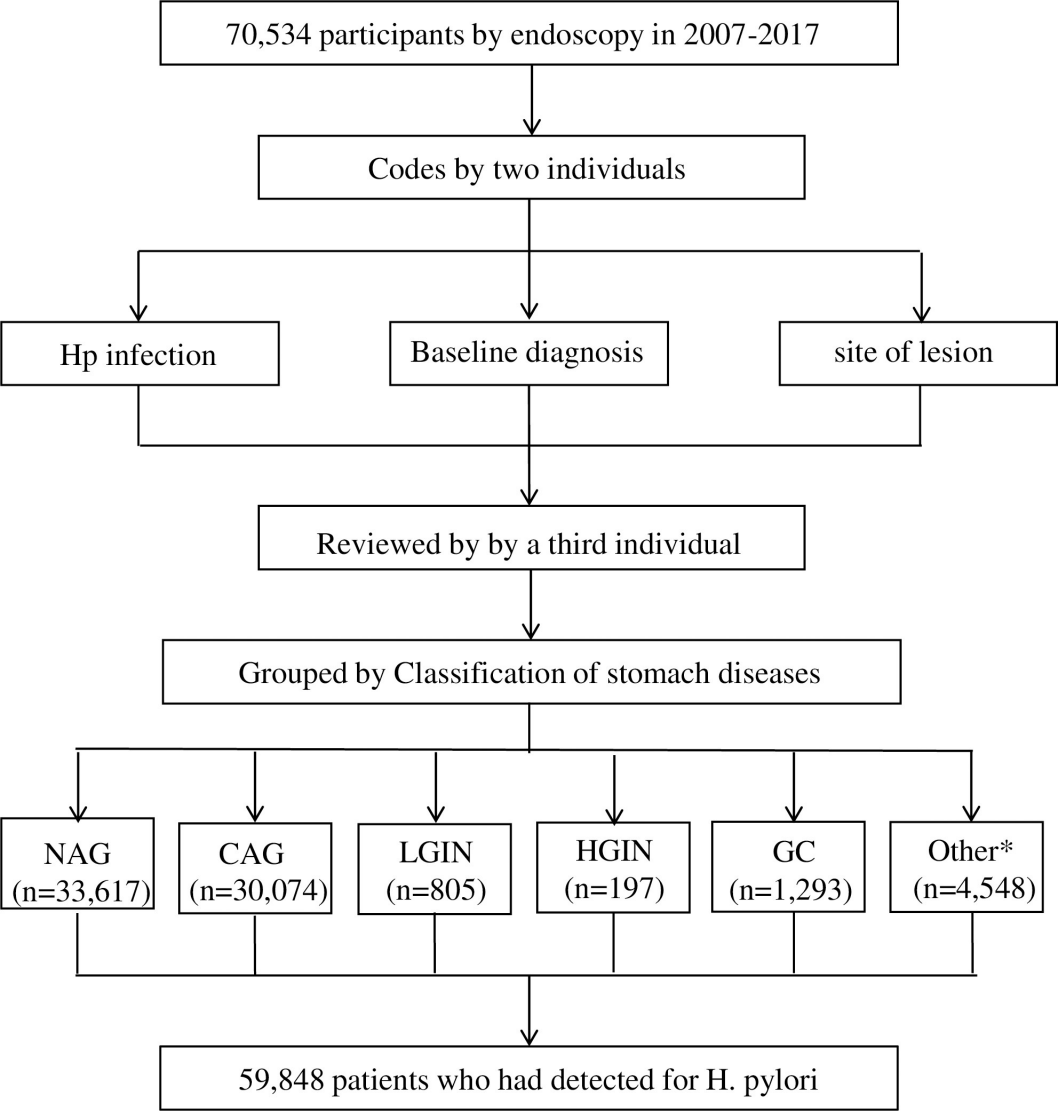
Patients in the overall sample (N = 70,534). A total of 70,534 cases of patients aged 18–94 (13,962 was excluded with multiple-visit endoscopic biopsy (n = 36,401) in total 98,241) who underwent gastroscopic biopsy for the first time in the First Affiliated Hospital of School of Medicine, Shihezi University from November 7, 2007 to December 31, 2017 were selected. Among them, 33,617 were non-atrophic gastritis (NAG) cases, 30,074 were chronic atrophic gastritis (CAG) cases, 805 were low-grade gastrointestinal intraepithelial neoplasia (LGIN) cases, 197 were high-grade gastric intraepithelial neoplasia (HGIN) cases, and 1293 were gastric cancer (GC) cases. *4548 were other diseases: lymphoma, gastric polyps, neuroendocrine neoplasm, liomyoma, gastric stromal tumor. Among them, 59,848 patients were detected for H. pylori infection.

A total of 70,534 cases aged 18–94 (36,401 biopsied cases from patients with multiple visits were excluded from a total of 106,935 biopsied cases) who underwent gastroscopic biopsy for the first time in our First Affiliated Hospital of School of Medicine, Shihezi University from November 2007 to December 2017 were included for analyses. Among them, 33,617 were non-atrophic gastritis (NAG) cases, 30,074 were chronic atrophic gastritis (CAG) cases, 805 were low-grade gastrointestinal intraepithelial neoplasia (LGIN) cases, 197 were high-grade gastric intraepithelial neoplasia (HGIN) cases, and 1,293 were gastric cancer (GC) cases. Among 70,534 cases, 59,848 cases were tested for H. pylori infection (Fig 1).

### Research methods

Biopsy was performed in accordance with the biopsy guidelines [14]. All biopsied specimens taken from visible lesions during endoscopy were fixed with 10% formalin, embedded with paraffin, sectioned (4μm thick), and stained with the hematoxylin and eosin method (HE). Histological sections from all gastric tissues collected for 10 years were diagnosed independently by two senior pathologists. Biopsies with no diagnostic consensus between the two pathologists were reviewed by a third senior pathologist to reach a diagnostic consensus. Methylene blue staining method was used for Hp detection. Hp infection was graded based on the amount of Hp colonization in glandular cavity and the surface of glandular cavity (S1 Table) [15].

### Statistical analyses

We considered P values of <0.05 to be statistically significant. All analyses were conducted using IBM SPSS statistical software (version 22.0). Chi-square (χ^2^) test was used to analyze differences between clinicopathological variables. Spearman’s rank correlation was used to identify correlations among preoperative and/or postoperative variables.

### Histological diagnosis categories

Gastric lesions were classified according to internationally accepted guidelines [8] for the diagnosis of NAG, CAG [16], LGIN, HGIN, and GC (S1 Fig). We assigned numerical codes to each diagnostic category following the Correa cascade: 1 = NAG, 2 = MAG, 3 = LGIN, 4 = HGIN, and 5 = GC. In any case with multiple biopsies, the most advanced lesion was considered as the histological diagnosis for that case.

### Ethics statement

Ethical approval was obtained from the Institutional Ethics Review Board (IERB) of the First Affiliated Hospital of School of Medicine, Shihezi University (No. 2018-067-01). The IERB waived the need for patient consents due to anonymous analyses of the data and confidentiality and anonymity in the handling and publication of patients’ tissues. Standard University Hospital Guidelines in accordance with the Declaration of Helsinki were followed in this study.

## Results

### Baseline characteristics of patients

There were 34,761 males and 35,773 females, accounting for 49.28% and 50.72% of the total cases, respectively. Expect for NAG patients, the detection rates of Hp infection and other stomach diseases (CAG → GC) were higher in male than those in female (*P* < 0.001). The number of males in Hp infection (13189, 43.90%), CAG (15322, 44.08%), LGIN (501, 1.44%), HGIN (149, 0.43%), and GC (992, 2.85%) were 13%, 4%, 65%, 210%, and 230% more than that of females, respectively; The detection rates of LGIN, HGIN, and GC in males were 1.44%, 0.43%, and 2.85%, respectively, while those in females were 0.85%, 0.13%, and 0.84%, respectively. The detection rates of LGIN and HGIN were lower than that of GC in males (*Pam* < 0.001, *Pcm* = 0.036), and there was no significant difference in the detection rates of LGIN, HGIN, and GC among females (*P* > 0.05) (Table 1).

**Table 1:** Differences in distribution of 70,534 gastroscopic biopsied patients between males and females.

	Total		Male		Female		χ^2^
	n	%	n	%	n	%	
NAG	33,617	47.66	16,467	47.37	17,150	47.94	2.29
CAG	30,074	42.64	15,322	44.08	14,752	41.24	58.15***
LGIN	805	1.14^a,b^	501	1.44^am,bm^	304	0.85^af,bf^	54.66***
HGIN	197	0.28^c^	149	0.43^cm^	48	0.13^cf^	54.89***
GC	1,293	1.83	992	2.85	301	0.84	396.73***
Other Diseases	4,548	6.45	1,330	3.83	3,218	9.00	781.03***
Total	70,534	100	34,761	100	35,773	100	1263.45***

Note: NAG = non-atrophic gastritis, CAG = chronic atrophic gastritis, LGIN = Low-grade gastric intraepithelial neopla sia, HGIN = High-grade gastric intraepithelial neoplasia, GC = gastric cancer. Other Diseases = lymphoma, gastric polyps, neuroendocrine neoplasm, liomyoma, gastric stromal tumor, etc.

***P < 0.001.

^am,af, a^Indicates comparison between LGIN and GC for male, female and both sex respectively (**P**^**am**^**<0.001**, P^af^ = 0.107 and **P**^**a**^**<0.001**); ^bm,bf,b^depicts comparison between LGIN and HGIN for male, female and both sex respectively (P^bm^ = 0.139, P^bf^ = 0.521 and P^b^ = 0.131); ^c,cm,cf^ refers to the comparison between HGIN and GC for male, female and both sex respectively (**P**^**cm**^ **= 0.036**, P^cf^ = 0.523 and P^c^ = 0.055).

### Characteristics of 1,293 gastric cancer patients

There were 1,293 GC cases (male: female ratio = 3: 1) (*P* < 0.001), with 1,251 cases of gastric adenocarcinoma (GAC) and 31 cases of gastric squamous cell carcinoma (GSCC) (GAC: GSCC 40:1). There was no significant difference in the number of adenocarcinoma and squamous cell carcinoma between males and females in gastric cancer (S2 Table).

### Patients with and without Hp infection

The number of males with Hp infection was significantly different from that of females in Hp infection, and the positive detection rate of Hp infection in males (13,189 cases, 43.90%) was higher than that in females (11,670 cases, 39.16%) (*P* < 0.001). Moreover, the positive detection rate of Hp infection decreased with age (*P* < 0.001) (Table 2). We found that the positive detection rate of Hp infection in atrophic gastritis patients (11,655 cases, 46.48%) was higher than that in non-atrophic gastritis patients (12,696 cases, 38.47%). The positive rate of Hp infection was statistically different among gastric diseases (P < 0.001). As shown in Table 2, a and b showed that the positive rate of Hp infection was lower than that of CAG in NAG and LGIN. # indicated that the positive rate of Hp infection in GIN was lower than that in GC, and there was a statistical difference. c and d showed that there was no statistical difference between LGIN and HGIN as well as between HGIN and GC (P>0.05). According to a study performed in 2015 [17], 671 cases (34.57%) of GC related to Hp infection and 1,270 cases (65.43%) of GC related to other factors in the process of gastritis and GIN eventually progressing to gastric cancer in the next 20 years (S3 Table).

**Table 2:** Comparisons of basic characteristics in patients with and without infection H. pylori.

Characteristics	H. pylori infection			χ^2^		p value
	yes	no	total			
Gender (n(%))						
Male	13,189(43.90)	16,857(56.10)	30,046 (100)	193.2		<0.001
Female	11,670(39.16)	18,132(60.84)	29,802 (100)			
Total	24,859 (41.54)	34,989 (58.46)	59,848 (100)			
Classification of Diseases (n(%))						
CAG	11,655 (46.48)	13,418 (53.52)	25,073 (100)		511.91	<0.001
LGIN	236 (33.01)	479 (66.99)	715 (100)	50.82*^b^		
HGIN*^$^	49 (29.52)	117 (70.48)	166 (100)	0.749^c^		
GC*^#^	223 (25.03)	668 (74.97)	891 (100)	1.476^d^		
Total	24,859 (41.54)	34,989 (58.46)	59,848 (100)			
Age group (n(%))						
18–20	84 (48.84)	88 (51.16)	172 (100)	980.91		<0.001
20–24	411 (51.70)	384 (48.30)	795 (100)			
25–29	767 (51.72)	716 (48.28)	1,483 (100)			
30–34	1,205 (48.26)	1,292 (51.74)	2,497 (100)			
35–39	2,272 (46.88)	2,574 (53.12)	4,846 (100)			
40–44	3,965 (45.03)	4,840 (54.97)	8,805 (100)			
45–49	4,732 (45.92)	5,573 (54.08)	10,305 (100)			
50–54	3,762 (43.52)	4,882 (56.48)	8,644 (100)			
55–59	2,431 (39.29)	3,756 (60.71)	6,187 (100)			
60–64	1,822 (36.54)	3,164 (63.46)	4,986 (100)			
65–69	1,421 (33.26)	2,852 (66.74)	4,273 (100)			
70–74	1,228 (30.22)	2,835 (69.78)	4,063 (100)			
75–79	569 (27.72)	1,484 (72.28)	2,053 (100)			
80–84	153 (26.02)	435 (73.98)	588 (100)			
≥85	37 (24.5)	114 (75.50)	151 (100)			
Total	24,859 (41.54)	34,989 (58.46)	59,848 (100)			

Note

*: P<0.001. a indicates comparison between NAG and CAG; b depicts comparison between CAG and LGIN; c refers to comparison between LGIN and HGIN; d indicates comparison between HGIN and GC; $ depicts comparison between Gastritis and GIN; # depicts comparison between GIN and GC.

### Correlation analysis

Hp infection was positively correlated with gastritis (r = 0.055, *P* < 0.001), while was negatively correlated with GIN and GAC (except for signet ring cell carcinoma, mucinous adenocarcinoma, etc) (*P* > 0.05). Further analysis showed that the detection ages of patients in gastritis and GIN were positively correlated with the severity of gastritis and GIN (*P* < 0.001), while the detection age of patients in gastric adenocarcinoma was negatively correlated with the degree of differentiation of gastric adenocarcinoma (r = -0.071, *P* = 0.023) (Table 3).

**Table 3:** Infection with helicobacter pylori and age in relation to the histological type of gastric diseases.

	n	H. pylori infection		r	p value	n	Age		r	p value
		(+~3+) n(%)	(-) n(%)				Mean	SD		
Gastritis										
NAG	33,003	12,696 (38.47)	20,307 (61.53)	0.055	<0.001	33,617	49.88	12.68	0.096	<0.001
CAG	25,073	11,655 (46.48)	13,418 (53.52)			30,074	52.56	13.31		
Intraepithelial neoplasia										
LGIN	715	236 (33.01)	479 (66.99)	-0.035	0.305	805	58.03	13.17	0.199	<0.001
HGIN	166	49 (29.52)	117 (70.48)			197	64.80	11.76		
adenocarcinoma of gastric (cell diff.)										
Well	9	3 (33.33)	6 (66.67)	-0.007	0.856	12	66.17	10.49	-0.071	0.023
Moderate	185	45 (24.32)	140 (75.68)			290	66.13	10.84		
Poor	513	125 (24.37)	388 (75.63)			724	64.04	12.13		

Note: In adenocarcinoma, except signet ring cell carcinoma, mucinous adenocarcinoma, etc

### Age groupings of patients with Hp infection, gastritis, GIN and GC

**Fig 2:**
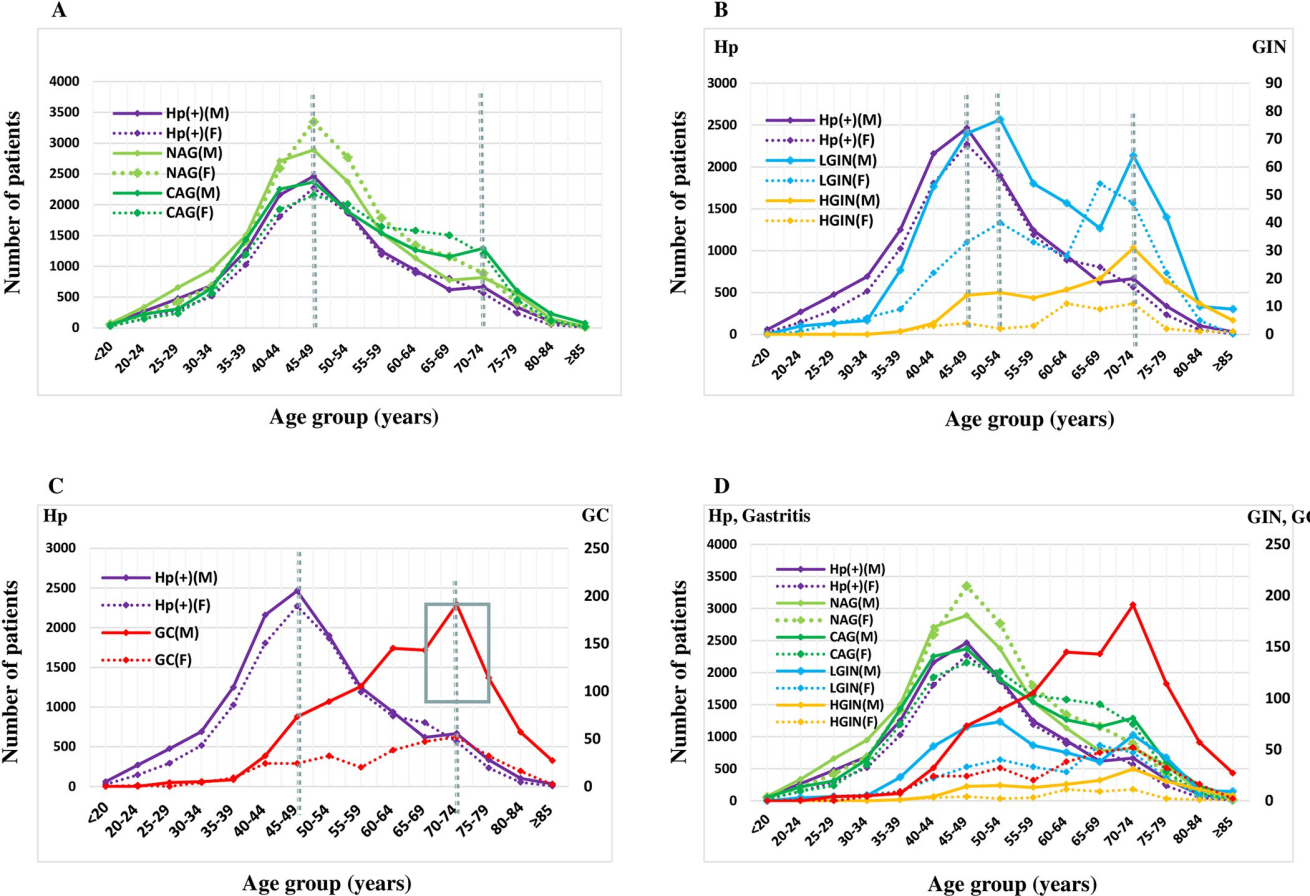
Age distributions of patients in Hp infection and gastric diseases detected by conventional gastroscopy. (A) age distributions of patients in gastritis and Hp infection; (B) age distributions of patients in GIN and Hp infection; (C) age distributions of patients in GC and Hp infection; (D) age distributions of patients in Hp infection, gastritis, GIN and GC; For males, The time spans (TS) from the first peak of Hp infection to the first peaks of gastric diseases varied dramatically depending on different gastric diseases: the overlapping peaks (0–5 years) for patients with gastritis; 5–10 years for patients with GINs, 15–20 years for patients with gastric cancer, respectively. It was interesting to note that the longest TS was 25–30 years from the first peak of Hp infection to the second peak of gastric cancer. The time spans (TS) from the ages of Hp infection patients at the second detection peak to the ages of gastritis, GIN, and GC patients at the second detection peak are 0–5 years. For females, The time spans (TS) from the first peak of Hp infection to the first peaks of gastric diseases varied dramatically depending on different gastric diseases: the overlapping peaks (0–5 years) for patients with gastritis; 5–10 years for patients with LGIN, 15–20 years for patients with HGIN, 5–10 years for patients with gastric cancer, respectively. It was interesting to note that the longest TS was 25–30 years from the first peak of Hp infection to the second peak of gastric cancer. However, there were also differences in time intervals from Hp infection at the second detection peak to the second peak of gastritis (0–5 years), GIN (5–10 years), and GC (5–10 years), respectively.

Twenty-three LGIN and fourteen GC patients were detected at ages 20–34, but no HGIN patients were detected (Table 4). We found that the ages of patients who were detected having Hp infection, gastritis, GIN, and GC had two prevalence peaks with one at 45–49 years and the other at 70–74 years, respectively. Among them, the detected number of patients with gastritis and LGIN was higher in patients aged 45–49 than those in patients aged 70–74. The detection number of HGIN and GC in patients aged 70–74 were higher than those in patients aged 45–49 (Fig 2). The age-related twin detection peaks for GC existed in both males and females with the first peak at 50–54 years and the second peak at 70–74 years. As shown in S4 Table, it was clear that the age-related twin prevalence peaks in female GC patients were more obvious than in male GC patients.

**Table 4:** Distribution of gastroscopic biopsied patients for Hp infection, gastritis, GIN and gastric cancer by age (years).

Age group	Hp (+~3+)		NAG		CAG		LGIN		HGIN		GC	
	n	%	n	%	n	%	n	%	n	%	n	%
18–20	84	0.34	124	0.37	80	0.27	_	_	_	_	_	_
20–24	411	1.65	542	1.61	366	1.22	4	0.5	_	_	1	0.08
25–29	767	3.09	1,076	3.2	544	1.81	8	0.99	_	_	4	0.31
30–34	1,205	4.85	1,623	4.83	1,209	4.02	11	1.37	_	_	9	0.7
35–39	2,272	9.14	2,915	8.67	2,623	8.72	32	3.98	2	1.02	16	1.24
40–44	3,965	15.95	5,297	15.76	4,176	13.89	75	9.32	7	3.55	56	4.33
**45–49**	**4,732**	**19.04**	**6,237**	**18.55**	**4,521**	**15.03**	**105**	**13.04**	**18**	**9.14**	**97**	**7.50**
50–54	3,762	15.13	5,141	15.29	3,899	12.96	117	14.53	17	8.63	121	9.36
55–59	2,431	9.78	3,324	9.89	3,182	10.58	87	10.81	16	8.12	125	9.67
60–64	1,822	7.33	2,472	7.35	2,842	9.45	75	9.32	27	13.71	183	14.15
65–69	1,421	5.72	1,932	5.75	2,657	8.83	92	11.43	29	14.72	190	14.69
**70–74**	**1,228**	**4.94**	**1,705**	**5.07**	**2,484**	**8.26**	**111**	**13.79**	**42**	**21.32**	**243**	**18.79**
75–79	569	2.29	957	2.85	1,054	3.5	64	7.95	21	10.66	146	11.29
80–84	153	0.62	233	0.69	343	1.14	15	1.86	12	6.09	73	5.65
≥85	37	0.15	39	0.12	94	0.31	9	1.12	6	3.05	29	2.24
Total	24,859	100	33,617	100	30,074	100	805	100	197	100	1,293	100

### The numbers of patients with Hp infection, gastritis, GIN and GC diagnosed at different ages

In males, the ages of patients in Hp infection and gastritis at the first detection peaks were 45–49 years old, and those at the second detection peaks were 70–74 years old; the ages of patients in LGIN and HGIN at the first detection peaks were 50–54 years old, and those at the second detection peaks were 70–74 years old; the ages of GC patients at the first detection peak were 60–64 years old, and those at the second detection peak were 70–74 years old. The time span (TS) from the first peak of Hp infection to the first peaks of different gastric diseases varied dramatically depending on actual gastric diseases: the overlapping peaks (0–5 years) for patients with gastritis; 5–10 years for patients with GINs, 15–20 years for patients with gastric cancer, respectively. It was interesting to note that the longest TS was 25–30 years, i.e., from the first peak of Hp infection to the second peak of gastric cancer. The TSs from the ages of patients with Hp infection at the second detection peak to the ages of patients with gastritis, GIN, and GC at the second detection peak were 0–5 years (Fig 2, S2 and S3 Figs).

In females, the ages of patients in Hp infection and gastritis at the first detection peaks were 45–49 years old, and those at the second detection peaks were 65–69 years old. The ages of LGIN patients at the first detection peak were 50–54 years old, and those at the second detection peak were 65–69 years old. The ages of HGIN patients at the first detection peak were 60–64 years old, and those at the second detection peak were 70–74 years old. The ages of GC patients at the first detection peak were 50–54 years old, and those at the second detection peak were 70–74 years old. The TSs from the first peak of Hp infection to the first peaks of gastric diseases varied dramatically depending on different gastric diseases: the TS was 0–5 years for patients with gastritis; 5–10 years for patients with LGIN, 15–20 years for patients with HGIN, and 5–10 years for patients with gastric cancer, respectively. It was interesting to note that the longest TS was 25–30 years from the first peak of Hp infection to the second peak of gastric cancer. However, there were also differences in time intervals from the second peak of Hp infection to the second peak of gastritis (0–5 years), GIN (5–10 years), and GC (5–10 years), respectively (Fig 2, S2 and S3 Figs).

## Discussion

More than 30 years ago, there were high incidences of GC in Japan and South Korea. Since mandatory gastroscopy screening had been carried out in the 1980s [3], the 5-year survival rates of gastric cancer in Japan and Korea had increased to 60.3% and 68.9%, respectively [18]. However, the detection rate of early gastric cancer in China has been less than 10% and the 5-year survival rate is only 35.9% [18, 19], a significant gap difference with developed countries in the world. Gastric cancer has seriously threatened the health and life of Chinese people and resulted in huge economic burden for individuals and the country [20]. This is the first large-scale retrospective study in Shihezi, a city with multi-ethnic populations in western China [21].

In this study, expect for NAG patients, the number of males in gastric diseases (CAG → GC) was more than that of females. The positive detection rate in males with Hp infection was higher than that in females (1.12: 1), which was consistent with the high detection rates of CAG, LGIN, HGIN, and GC in males. The patients with CAG had higher Hp infection rates than those with NAG, and the detection rates of Hp infection decreased along with aggravated gastric disease. We favor the hypothesis that Hp infection may cause gastric cancer primarily via CAG, not NAG. This hypothesis is supported by our observations that the prevalence of NAG in male patients is not higher than that in female patients who have lower prevalence of Hp infection as well as GC than male patients. Thus, only a small proportion of females infected by Hp developed more severe gastric diseases and GC. Our study has also found that the number of GC patients diagnosed in males was 3.3 times higher than that in females. According to GLOBOCAN 2018, the incidence of GC in males is more than 2 fold higher than in women in some countries [1] in keeping with our observations.

Among males, the detection rate of HGIN is lower than that of LGIN and that of GC. The same is true among females. LGIN and GC can be detected in patients aged 20–34 years, among whom HGIN is not detected. According to the Correa cascade hypothesis, the number of patients with gastric diseases or the detection rate of gastric diseases decreases gradually with the aggravation of gastric diseases. However, this is not consistent with the detection rate of HGIN in our dataset. Thus, we hypothesize that HGIN may be a short pathological process which may be easily missed in routine clinics [22]. In addition, a data released by the World Health Organization (WHO) shows that severe dysplasia [8, 23] (the WHO recently classified severe dysplasia as HGIN) needs a time span from 4 months to 1.5 years to develop GC [24], which verifies that the process from HGIN to GC is a short-term process, particularly for adolescent HGIN patients who may develop GC in half a year. These observations may explain why LGIN and GC, not HGIN, can be detected in patients at ages 20–34. Therefore, the optimal follow-up interval should be determined based on the observations to avoid missing diagnosis of HGIN which is precancerous to GC.

The younger the patients, the more Hp infection and gastritis are detected but to the contrary, the fewer GIN and GC detected. With increased age, the numbers of GIN and GC patients increase and the number of Hp infection and gastritis patients decrease. The dynamic trend of Hp infection with patients’ age is consistent with that of gastritis with patients’ age, but in contrary to the dynamic trends of GIN and GC with patients’ ages. This is consistent with the observed trend that the detection rate of Hp infection decreases with aggravated gastric diseases (LGIN → HGIN → GC) [25, 26].

Some studies have shown that Hp infection can promote the development of NAG to CAG, GIN and GC [27, 28]. In this study, the magnitude or severity of Hp infection in GC patients is significantly lower than that in gastritis patients, and the gastric microbial diversity in GC patients is also significantly lower than that in chronic gastritis patients. Furthermore, Hp infection exists in the gastric carcinoma microbiota as a low abundant or absent genus [25]. It may be that gastritis with intestinal metaplasia (IM) and GIN are the body’s self-protection performance with the aggravation of gastric diseases. Accordingly, the gastric micro-environment is not suitable for Hp to settle here [29], which reduces the further damage of Hp to the gastric mucosa. Overall, these observations let us to hypothesize that Hp infection may be an early event and the early eradication of Hp infection may be therefore a vital remedy for effective prevention of ultimate development of GC.

One major finding of this study is that, for the first time, we have observed an interesting phenomenon that there are age-related twin-peak presentation profiles (TPPs) for all gastric diseases from Hp infection, gastritis to advanced diseases, including GC, with different time spans (TS) between the two peaks. As shown in Fig 2, the first occurring peaks of early diseases, such as Hp infection and gastritis, appear to be overlapped but they occur significantly earlier than the first occurring peaks of more severe diseases, such as GIN and GC. Further notification is that more patients with GIN and GC are concentrated at the second peak than those at the first peak. This observed phenomenon warrants further epidemiological investigations to explore the underlying mechanism(s) causing the presentation of TPPs in gastric diseases.

As shown in Fig 2, from the ages of patients with Hp infection at the first peak to the ages of patients with gastric diseases at the first peaks, the TSs are longer than those from the ages of patients with Hp infection at the second peak to the ages of patients with gastric diseases at the second peaks. These observations suggest that a second Hp infection may have a greater impact on the aggravation of gastric diseases than the first Hp infection. Accordingly, clinical attentions should be paid to the second Hp infection to reduce the risk of developing gastric diseases especially GC.

Gastritis, GIN and GC patients are found to have TPPs. The ages of males and females in gastric diseases at the first detection peak are 45–49 years old. Interestingly, the ages of females in gastritis and GIN at the second peak are 5–10 years earlier than the ages of males (Fig 2). These observations suggest that, in clinical screening for GC, the cut-off ages should be rationally differentiated for males and females respectively, and accordingly, clinical screening for GC for males should be extended 5–10 years beyond the ages for females [30]. Based on evidence provided in this large dataset, we recommend that screening strategy for early detection of GC be extended to 79 year-olds for males.

In conclusion, by analyzing a large dataset of 70,534 routine cases with histologically diagnosed gastroscopic biopsies, we have, for the first time, observed the “twin-peak prevalence profile” or TPP for all gastric diseases analyzed, including Hp infection, gastritis, GIN and GC, respectively. Based on the observations presented, we recommend that screening for gastric cancer be extended to 79 year-olds for males, at least in China.

## Supporting information

S1 Table Grades of H *pylori* infection. (DOC)

S2 Table Characteristics of 1293 gastric cancer patients enrolled in stomach biopsy study. (DOCX)

S3 Table Estimated gastric cancer (GC) cases for patients with Hp infection correlating with a histologic stage in Correa’s cascade. (DOCX)

S4 Table Distribution of gastroscopic biopsied patients for Hp Infection, Gastritis, GIN and Gastric Cancer by age (years) between males and females. (DOC)

S1 Fig Histological micrographs of gastric diseases. (TIF)

S2 Fig Age distribution of patients in Hp infection and gastritis. (TIF)

S3 Fig Age distribution of patients in Hp infection, GIN and GC. (TIF)
