# Supplementary material for: Age-related twin-peak prevalence profiles of H. pylori infection, gastritis, GIN and gastric cancer: Analyses of 70,534 patients with gastroscopic biopsies
Source: PLoS One. 2022 Jul 21;17(7):e0265885. doi: 10.1371/journal.pone.0265885 (PMC9302749; doi:10.1371/journal.pone.0265885)
Supplement: S1 Table — (DOC) [file pone.0265885.s001.doc]

| **S1 Table. Grades of H** pylori infection | | |
| --- | --- | --- |
| Grades | the amount of H pylori colonization | |
|  | Surface of glandular cavity | in the glands |
| - | absent | absent |
| + | few | absent |
| ++ | vary in amount | few, scattered |
| +++ | vary in amount | Large quantity |
