# Supplementary material for: Age-related twin-peak prevalence profiles of H. pylori infection, gastritis, GIN and gastric cancer: Analyses of 70,534 patients with gastroscopic biopsies
Source: PLoS One. 2022 Jul 21;17(7):e0265885. doi: 10.1371/journal.pone.0265885 (PMC9302749; doi:10.1371/journal.pone.0265885)
Supplement: S2 Table — (DOCX) [file pone.0265885.s002.docx]

| **S2 Table. Characteristics of 1293 gastric cancer patients enrolled in stomach biopsy study** | | | | | | | | | | | | |
| --- | --- | --- | --- | --- | --- | --- | --- | --- | --- | --- | --- | --- |
| Classification | Total | |  | male | |  | female | | Ratio* | χ |  | P value |
|  | n | % |  | n | % |  | n | % |  |  |  |  |
| SCC | 31 | 2.40 |  | 25 | 2.52 |  | 6 | 1.99 | 4.17: 1 | 0.274 |  | 0.601 |
| AC | 1,251 | 96.75 |  | 961 | 96.88 |  | 290 | 96.35 | 3.31: 1 |  |  |  |
| Total | 1,293 | 100 |  | 992 | 100 |  | 301 | 100 | 3.30: 1 |  |  |  |
| Note：SCC: squamous carcinoma; AC: adenocarcinoma. *: Male: Female. | | | | | | | | | | | | |
|  |  |  |  |  |  |  |  |  |  |  |  |  |
