# Supplementary material for: Age-related twin-peak prevalence profiles of H. pylori infection, gastritis, GIN and gastric cancer: Analyses of 70,534 patients with gastroscopic biopsies
Source: PLoS One. 2022 Jul 21;17(7):e0265885. doi: 10.1371/journal.pone.0265885 (PMC9302749; doi:10.1371/journal.pone.0265885)
Supplement: S3 Table — (DOCX) [file pone.0265885.s003.docx]

| **S3 Table. Estimated gastric cancer (GC) cases for patients with Hp infection correlating with a histologic stage in Correa’s cascade.** | | | | | | | | | | |
| --- | --- | --- | --- | --- | --- | --- | --- | --- | --- | --- |
| Characteristics | | *ratio of GC | H. pylori infection | | | | | | | |
|  |  |  | YES | |  | NO | |  | Total | |
|  |  |  | n | Estimated  cancer cases |  | n | Estimated  cancer cases |  | n | Estimated cancer cases |
|  |  |  |  | n (%) |  |  | n (%) |  |  | n (%) |
| NAG | |  | 12,696 | 177 (39.07) |  | 20,307 | 276 (60.93) |  | 33,003 | 453 (100) |
|  | with IM | **1: 39** | 2,042 | 52 (42.98) |  | 2,681 | 69 (57.02) |  | 4,723 | 121 (100) |
|  | without IM | **1: 85** | 10,654 | 125 (37.65) |  | 17,626 | 207 (62.35) |  | 28,280 | 332 (100) |
| CAG | |  | 11,655 | 256 (46.46) |  | 13,418 | 295 (53.54) |  | 25,073 | 551 (100) |
|  | with IM | **1: 39** | 4,101 | 105 (46.46) |  | 4,720 | 121 (53.54) |  | 8,821 | 226 (100) |
|  | without IM | **1: 50** | 7,554 | 151 (46.46) |  | 8,698 | 174 (53.54) |  | 16,252 | 325 (100) |
| LGIN | | **1: 19** | 236 | 12 (32.43) |  | 479 | 25 (67.57) |  | 715 | 37 (100) |
| HGIN | | **1: 19** | 49 | 3 (33.33) |  | 117 | 6 (66.67) |  | 166 | 9 (100) |
| GC | | **1: 1** | 223 | 223 (25.03) |  | 668 | 668 (74.97) |  | 891 | 891 (100) |
| Total | |  | 24,859 | **671(34.57)** |  | 34,989 | 1270 (65.43) |  | 59,848 | 1941 (100) |
| Note: IM, intestinal metaplasia; *Data are from this study (Incidence of gastric cancer among patients with gastric precancerous lesions: observational cohort study in a low risk Western population. BMJ 351: h3867). | | | | | | | | | | |
