# Supplementary material for: Age-related twin-peak prevalence profiles of H. pylori infection, gastritis, GIN and gastric cancer: Analyses of 70,534 patients with gastroscopic biopsies
Source: PLoS One. 2022 Jul 21;17(7):e0265885. doi: 10.1371/journal.pone.0265885 (PMC9302749; doi:10.1371/journal.pone.0265885)
Supplement: S4 Table — (DOC) [file pone.0265885.s004.doc]

| **S4 Table.** **Distribution of gastroscopic biopsied patients for Hp Infection, Gastritis, GIN and Gastric Cancer by age (years) between males and females** | | | | | | | | | | | | | | | | | |
| --- | --- | --- | --- | --- | --- | --- | --- | --- | --- | --- | --- | --- | --- | --- | --- | --- | --- |
| Age group | Hp (1+~3+) | |  | NAG | |  | CAG | |  | LGIN | |  | HGIN | |  | GC | |
|  | n | % |  | n | % |  | n | % |  | n | % |  | n | % |  | n | % |
| Both sex |  |  |  |  |  |  |  |  |  |  |  |  |  |  |  |  |  |
| <20 | 84 | 0.34 |  | 124 | 0.37 |  | 80 | 0.27 |  | _ | _ |  | _ | _ |  | _ | _ |
| 20-24 | 411 | 1.65 |  | 542 | 1.61 |  | 366 | 1.22 |  | 4 | 0.5 |  | _ | _ |  | 1 | 0.08 |
| 25-29 | 767 | 3.09 |  | 1,076 | 3.2 |  | 544 | 1.81 |  | 8 | 0.99 |  | _ | _ |  | 4 | 0.31 |
| 30-34 | 1,205 | 4.85 |  | 1,623 | 4.83 |  | 1,209 | 4.02 |  | 11 | 1.37 |  | _ | _ |  | 9 | 0.7 |
| 35-39 | 2,272 | 9.14 |  | 2,915 | 8.67 |  | 2,623 | 8.72 |  | 32 | 3.98 |  | 2 | 1.02 |  | 16 | 1.24 |
| 40-44 | 3,965 | 15.95 |  | 5,297 | 15.76 |  | 4,176 | 13.89 |  | 75 | 9.32 |  | 7 | 3.55 |  | 56 | 4.33 |
| **45-49** | **4,732** | **19.04** |  | **6,237** | **18.55** |  | **4,521** | **15.03** |  | **105** | **13.04** |  | **18** | **9.14** |  | **97** | **7.50** |
| 50-54 | 3,762 | 15.13 |  | 5,141 | 15.29 |  | 3,899 | 12.96 |  | 117 | 14.53 |  | 17 | 8.63 |  | 121 | 9.36 |
| 55-59 | 2,431 | 9.78 |  | 3,324 | 9.89 |  | 3,182 | 10.58 |  | 87 | 10.81 |  | 16 | 8.12 |  | 125 | 9.67 |
| 60-64 | 1,822 | 7.33 |  | 2,472 | 7.35 |  | 2,842 | 9.45 |  | 75 | 9.32 |  | 27 | 13.71 |  | 183 | 14.15 |
| 65-69 | 1,421 | 5.72 |  | 1,932 | 5.75 |  | 2,657 | 8.83 |  | 92 | 11.43 |  | 29 | 14.72 |  | 190 | 14.69 |
| **70-74** | **1,228** | **4.94** |  | **1,705** | **5.07** |  | **2,484** | **8.26** |  | **111** | **13.79** |  | **42** | **21.32** |  | **243** | **18.79** |
| 75-79 | 569 | 2.29 |  | 957 | 2.85 |  | 1,054 | 3.5 |  | 64 | 7.95 |  | 21 | 10.66 |  | 146 | 11.29 |
| 80-84 | 153 | 0.62 |  | 233 | 0.69 |  | 343 | 1.14 |  | 15 | 1.86 |  | 12 | 6.09 |  | 73 | 5.65 |
| ≥85 | 37 | 0.15 |  | 39 | 0.12 |  | 94 | 0.31 |  | 9 | 1.12 |  | 6 | 3.05 |  | 29 | 2.24 |
| Total | 24,859 | 100 |  | 33,617 | 100 |  | 30,074 | 100 |  | 805 | 100 |  | 197 | 100 |  | 1,293 | 100 |
| Males |  |  |  |  |  |  |  |  |  |  |  |  |  |  |  |  |  |
| <20 | 58 | 0.44 |  | 68 | 0.41 |  | 54 | 0.35 |  | _ | _ |  | _ | _ |  | _ | _ |
| 20-24 | 267 | 2.02 |  | 335 | 2.03 |  | 219 | 1.43 |  | 3 | 0.6 |  | _ | _ |  | _ | _ |
| 25-29 | 475 | 3.6 |  | 656 | 3.98 |  | 309 | 2.02 |  | 4 | 0.8 |  | _ | _ |  | 4 | 0.4 |
| 30-34 | 690 | 5.23 |  | 946 | 5.74 |  | 647 | 4.22 |  | 5 | 1 |  | _ | _ |  | 5 | 0.5 |
| 35-39 | 1,248 | 9.46 |  | 1,502 | 9.12 |  | 1,432 | 9.35 |  | 23 | 4.59 |  | 1 | 0.67 |  | 7 | 0.71 |
| 40-44 | 2,159 | 16.37 |  | 2,708 | 16.45 |  | 2,251 | 14.69 |  | 53 | 10.58 |  | 4 | 2.68 |  | 32 | 3.23 |
| **45-49** | **2,463** | **18.67** |  | **2,890** | **17.55** |  | **2,369** | **15.46** |  | **72** | **14.37** |  | **14** | **9.4** |  | **73** | **7.36** |
| 50-54 | 1,898 | 14.39 |  | 2,375 | 14.42 |  | 1,890 | 12.34 |  | 77 | 15.37 |  | 15 | 10.07 |  | 89 | 8.97 |
| 55-59 | 1,244 | 9.43 |  | 1,540 | 9.35 |  | 1,540 | 10.05 |  | 54 | 10.78 |  | 13 | 8.72 |  | 105 | 10.58 |
| 60-64 | 936 | 7.1 |  | 1,132 | 6.87 |  | 1,264 | 8.25 |  | 47 | 9.38 |  | 16 | 10.74 |  | 145 | 14.62 |
| 65-69 | 618 | 4.69 |  | 775 | 4.71 |  | 1,154 | 7.53 |  | 38 | 7.58 |  | 20 | 13.42 |  | 143 | 14.42 |
| **70-74** | **665** | **5.04** |  | **818** | **4.97** |  | **1,289** | **8.41** |  | **64** | **12.77** |  | **31** | **20.81** |  | **191** | **19.25** |
| 75-79 | 336 | 2.55 |  | 551 | 3.35 |  | 600 | 3.92 |  | 42 | 8.38 |  | 19 | 12.75 |  | 114 | 11.49 |
| 80-84 | 101 | 0.77 |  | 144 | 0.87 |  | 228 | 1.49 |  | 10 | 2 |  | 11 | 7.38 |  | 57 | 5.75 |
| ≥85 | 31 | 0.24 |  | 27 | 0.16 |  | 76 | 0.5 |  | 9 | 1.8 |  | 5 | 3.36 |  | 27 | 2.72 |
| Total | 13,189 | 100 |  | 16,467 | 100 |  | 15,322 | 100 |  | 501 | 100 |  | 149 | 100 |  | 992 | 100 |
| Females |  |  |  |  |  |  |  |  |  |  |  |  |  |  |  |  |  |
| <20 | 26 | 0.22 |  | 56 | 0.33 |  | 26 | 0.18 |  | _ | _ |  | _ | _ |  | _ | _ |
| 20-24 | 144 | 1.23 |  | 207 | 1.21 |  | 147 | 1 |  | 1 | 0.33 |  | _ | _ |  | 1 | 0.33 |
| 25-29 | 292 | 2.5 |  | 420 | 2.45 |  | 235 | 1.59 |  | 4 | 1.32 |  | _ | _ |  | _ | _ |
| 30-34 | 515 | 4.41 |  | 677 | 3.95 |  | 562 | 3.81 |  | 6 | 1.97 |  | _ | _ |  | 4 | 1.33 |
| 35-39 | 1,024 | 8.77 |  | 1,413 | 8.24 |  | 1,191 | 8.07 |  | 9 | 2.96 |  | 1 | 2.08 |  | 9 | 2.99 |
| 40-44 | 1,806 | 15.48 |  | 2,589 | 15.1 |  | 1,925 | 13.05 |  | 22 | 7.24 |  | 3 | 6.25 |  | 24 | 7.97 |
| **45-49** | **2,269** | **19.44** |  | **3,347** | **19.52** |  | **2,152** | **14.59** |  | **33** | **10.86** |  | **4** | **8.33** |  | **24** | **7.97** |
| 50-54 | 1,864 | 15.97 |  | 2,766 | 16.13 |  | 2,009 | 13.62 |  | 40 | 13.16 |  | 2 | 4.17 |  | 32 | 10.63 |
| 55-59 | 1,187 | 10.17 |  | 1,784 | 10.4 |  | 1,642 | 11.13 |  | 33 | 10.86 |  | 3 | 6.25 |  | 20 | 6.64 |
| 60-64 | 886 | 7.59 |  | 1,340 | 7.81 |  | 1,578 | 10.7 |  | 28 | 9.21 |  | 11 | 22.92 |  | 38 | 12.62 |
| 65-69 | 803 | 6.88 |  | 1,157 | 6.75 |  | 1,503 | 10.19 |  | 54 | 17.76 |  | 9 | 18.75 |  | 47 | 15.61 |
| **70-74** | **563** | **4.82** |  | **887** | **5.17** |  | **1,195** | **8.1** |  | **47** | **15.46** |  | **11** | **22.92** |  | **52** | **17.28** |
| 75-79 | 233 | 2.00 |  | 406 | 2.37 |  | 454 | 3.08 |  | 22 | 7.24 |  | 2 | 4.17 |  | 32 | 10.63 |
| 80-84 | 52 | 0.45 |  | 89 | 0.52 |  | 115 | 0.78 |  | 5 | 1.64 |  | 1 | 2.08 |  | 16 | 5.32 |
| ≥85 | 6 | 0.05 |  | 12 | 0.07 |  | 18 | 0.12 |  | _ | _ |  | 1 | 2.08 |  | 2 | 0.66 |
| Total | 11,670 | 100 |  | 17,150 | 100 |  | 14,752 | 100 |  | 304 | 100 |  | 48 | 100 |  | 301 | 100 |
