# Supplementary figures and images for: Age-related twin-peak prevalence profiles of H. pylori infection, gastritis, GIN and gastric cancer: Analyses of 70,534 patients with gastroscopic biopsies
Source: PLoS One. 2022 Jul 21;17(7):e0265885. doi: 10.1371/journal.pone.0265885 (PMC9302749; doi:10.1371/journal.pone.0265885)

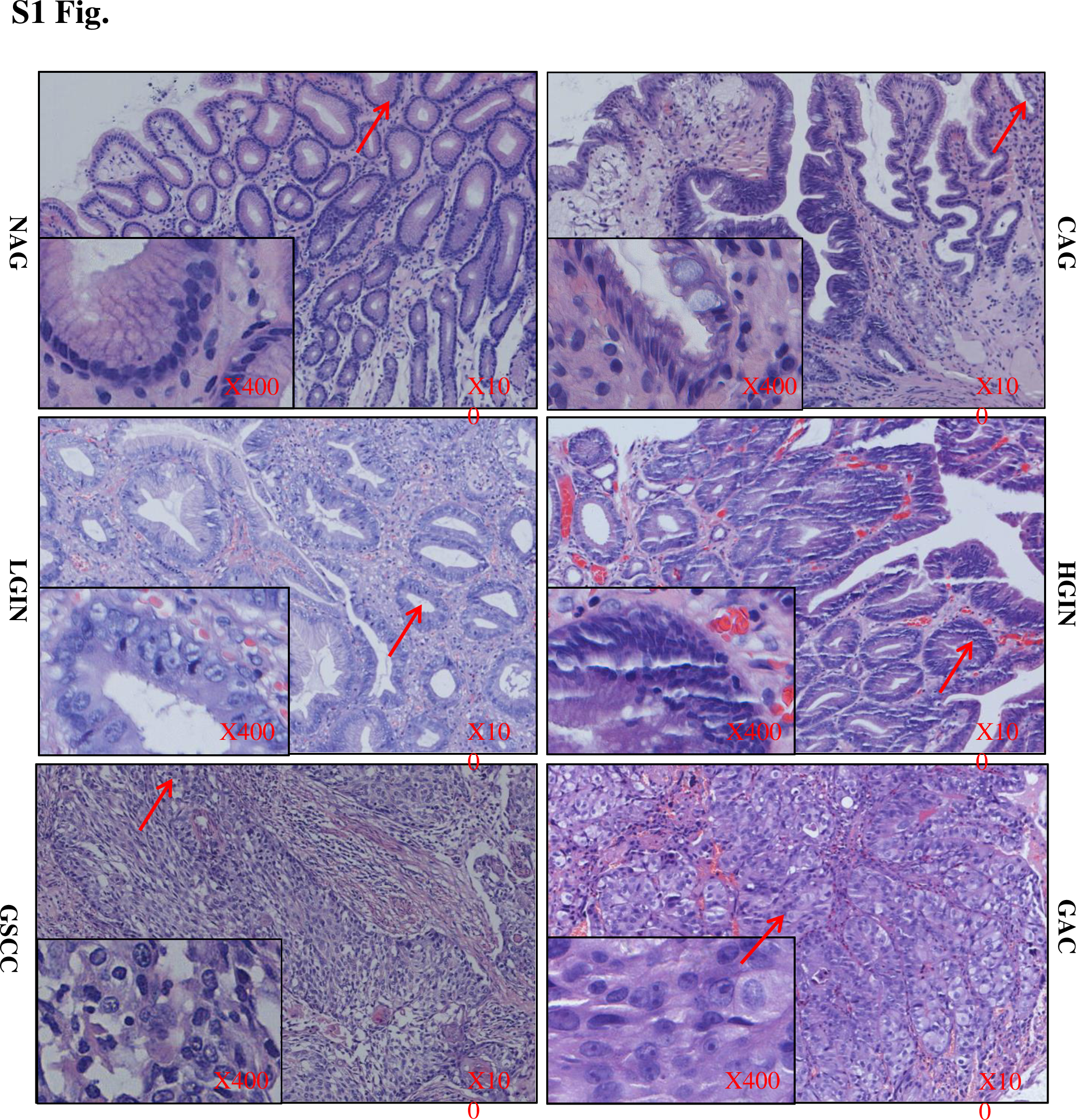

Supplement: S1 Fig — (TIF) [file pone.0265885.s005.tif]

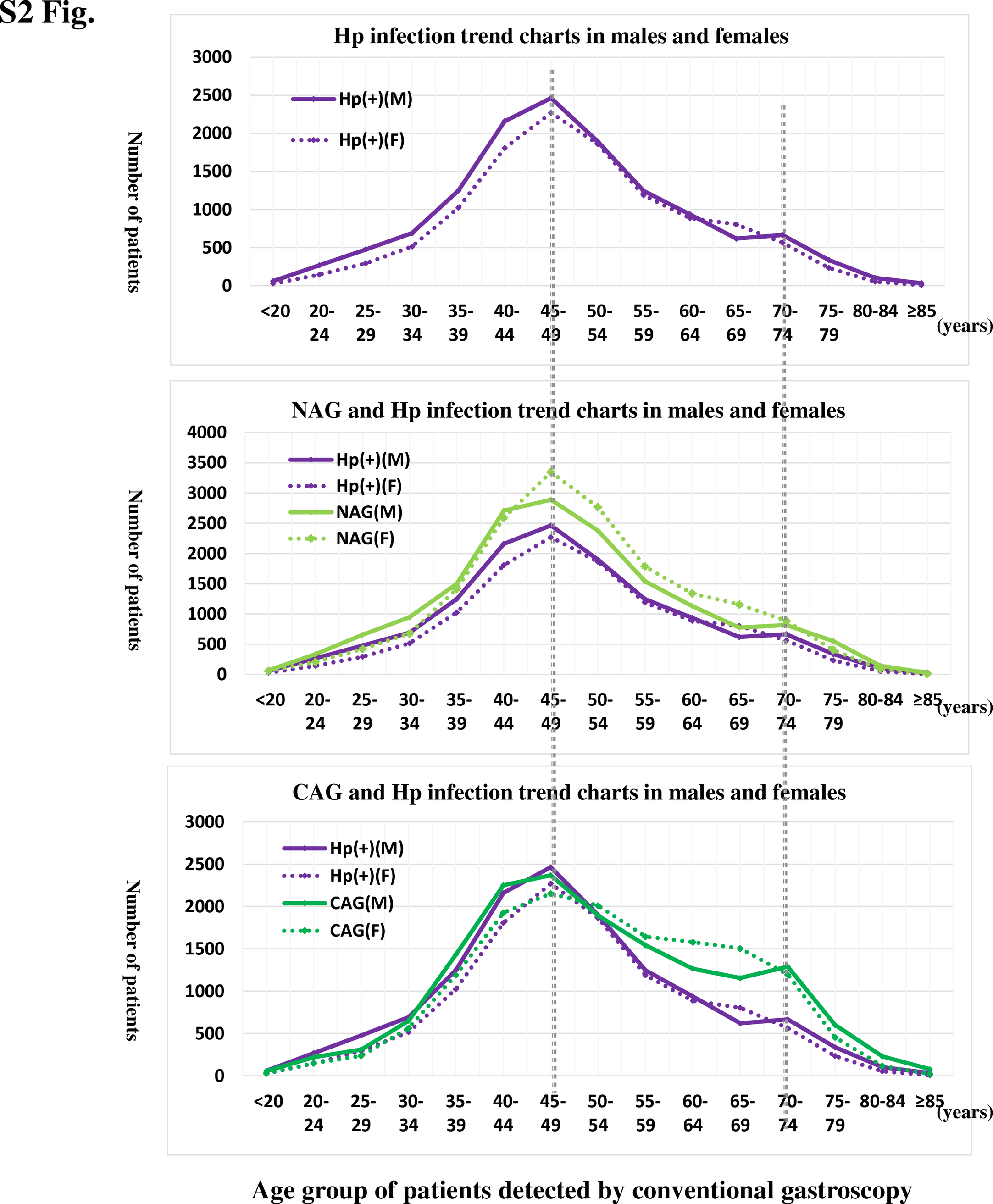

Supplement: S2 Fig — (TIF) [file pone.0265885.s006.tif]

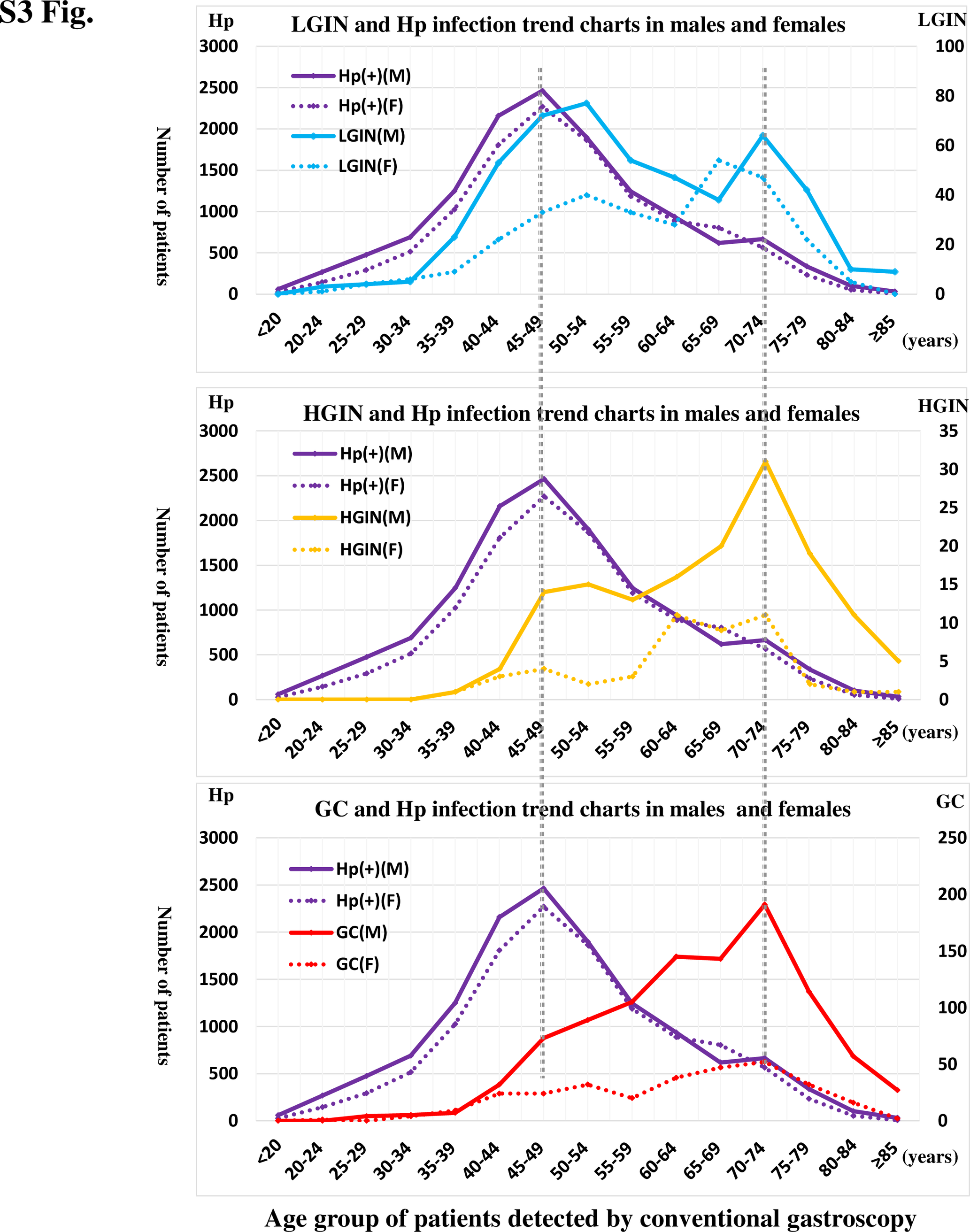

Supplement: S3 Fig — (TIF) [file pone.0265885.s007.tif]
